# Use of Cyanoacrylate (Superglue) for the Repair of Incidental Durotomy During Lumbar Spine Surgery: A Low-Cost Technique in a Resource-Limited Setting

**DOI:** 10.7759/cureus.110108

**Published:** 2026-06-02

**Authors:** Ali Elijah Usiholo, George A Akpede

**Affiliations:** 1 Neurosurgery, University of Benin Teaching Hospital, Benin, NGA

**Keywords:** cerebrospinal fluid leak, cyanoacrylate, incidental dural tear, lumbar spine surgery, resource-limited setting, superglue

## Abstract

Background

Incidental dural tears are a recognized complication of lumbar spine surgery and can lead to significant postoperative morbidity if not properly managed. Standard repair techniques often rely on specialized grafts and sealants that may be costly or unavailable in low-resource settings. This study reports our experience with the use of cyanoacrylate adhesive (superglue) for intraoperative repair of dural tears in a resource-limited environment.

Methods

We retrospectively reviewed patients who sustained incidental dural tears during lumbar spine surgery at a tertiary spine centre in a resource-limited setting between January 2022 and June 2025. All tears were identified intraoperatively and repaired immediately. In six cases, cyanoacrylate adhesive was used as part of the dural repair strategy. In two patients, lumbar fascia grafts were used to cover inaccessible tears and reinforced with Surgicel™ (Ethicon, Inc., Raritan, New Jersey) before application of cyanoacrylate adhesive. In four patients, autologous fat grafts and Surgicel™ were similarly used prior to cyanoacrylate application. Postoperative outcomes, including cerebrospinal fluid (CSF) leakage, wound infection, pseudomeningocele formation, and need for reoperation, were reviewed.

Results

Among 68 patients who underwent lumbar spine surgery, nine (13.2%) sustained intraoperative dural tears, and six of these were managed using cyanoacrylate-assisted repair. Watertight closure was achieved intraoperatively in all cases. One patient developed a mild superficial wound infection that resolved with antibiotics, while another developed a pseudomeningocele following revision surgery that was managed conservatively. There were no cases of persistent CSF leakage, meningitis, or reoperation.

Conclusion

Cyanoacrylate-assisted repair appeared to provide a feasible and low-cost adjunct for dural repair in this limited case series. When used in conjunction with autologous grafts and Surgicel™, satisfactory short-term outcomes were observed without major complications. Further prospective studies are needed to better establish long-term safety and effectiveness.

## Introduction

Incidental dural tear is one of the most frequently encountered complications of lumbar spine surgery, with reported incidence rates ranging from 1% to 17% [1-3]. Although most cases can be successfully repaired intraoperatively, missed or inadequately sealed tears may result in cerebrospinal fluid (CSF) leakage, wound infection, meningitis, pseudomeningocele, or prolonged hospitalization [4-6]. In high-resource settings, commercially available collagen matrices, fibrin sealants, and synthetic polymer-based adhesives are often employed to reinforce dural repair and minimize CSF leakage [6-8]. However, such materials are often unavailable or prohibitively expensive in low- and middle-income countries (LMICs).

Cyanoacrylate-based adhesives, commonly marketed as superglue, are synthetic monomers that rapidly polymerize upon contact with tissue moisture, forming a strong seal [9]. These adhesives have been used in various surgical disciplines for haemostasis, skin closure, and selected tissue reinforcement applications, offering a potentially low-cost and accessible alternative where proprietary sealants are unavailable [10]. However, published data regarding their use in dural repair remain limited, particularly in sub-Saharan Africa.

This study describes our experience with cyanoacrylate-assisted repair of incidental dural tears during lumbar spine surgery in a resource-limited setting and evaluates the feasibility, safety considerations, and short-term postoperative outcomes associated with this technique.

## Materials and methods

Study design and setting

This was a retrospective observational study conducted at Zimran Medical Centre, Benin City, Nigeria, a private tertiary referral centre for spine surgery. The study covered a three-and-a-half-year period from January 2022 to June 2025. Ethical approval for the study was obtained from the institutional review committee, and patient confidentiality was maintained throughout the review process.

Patient selection

All patients who underwent lumbar spine surgery during the study period were included in the initial review. Patients with intradural pathology requiring intentional durotomy and those with traumatic dural tears secondary to spinal fractures were excluded from the analysis. Data were extracted from operative records, anaesthetic notes, and postoperative follow-up documentation.

Data collection and variables

Demographic information, including age and sex, along with operative variables, such as diagnosis, number of decompression levels, estimated blood loss, duration of surgery, and intraoperative findings, were obtained.

For patients who sustained incidental dural tears, additional variables recorded included tear location and size, repair technique employed (suture, graft, or sealant), use of cyanoacrylate adhesive, and both intraoperative and postoperative outcomes.

Postoperative outcomes assessed included CSF leakage, wound infection, pseudomeningocele formation, neurological deterioration, and the need for reoperation. No formal standardized outcome assessment tool was employed; outcomes were assessed clinically based on predefined postoperative parameters.

Statistical analysis

Given the descriptive nature of this retrospective case series and the small sample size, formal comparative statistical analysis was not performed. Data were summarized using descriptive statistics, including frequencies, percentages, means, and standard deviations, where appropriate.

Surgical technique

All procedures were performed under regional (combined spinal and epidural) anaesthesia, with patients positioned prone. Following exposure of the affected lumbar segments, decompression was achieved through standard laminectomy or laminotomy, with or without instrumented fusion depending on the indication.

When a dural tear was identified intraoperatively, the site was gently cleaned and temporarily covered with a cottonoid patty. In cases where the dural edges were clearly visualized and accessible, a small autologous fat graft harvested from the subcutaneous tissue was applied over the defect and covered with Surgicel™ (Ethicon, Inc., Raritan, New Jersey).

Ethyl cyanoacrylate adhesive (OKe Bond™, Zhejiang Jiuerjiu Chemicals Co. Ltd., Yiwu, China) was then used as a reinforcing sealant. Approximately two to three drops were applied in most repairs, while one patient required four drops because of defect characteristics. To minimize spread and reduce potential thermal or inflammatory injury to surrounding tissues, damp cottonoid patties were placed over adjacent exposed dura and soft tissues during application. The adhesive was applied over the Surgicel-covered repair rather than directly onto neural structures. Polymerization was visually observed to occur completely before proceeding with wound closure, and care was taken throughout the procedure to avoid direct neural exposure.

For larger, inaccessible, lateral, anterior, or friable tears where direct coverage was more difficult, a lumbar fascia graft harvested from the operative field was used to patch the defect. This was similarly covered with Surgicel™ and reinforced with cyanoacrylate adhesive. A Valsalva manoeuvre was then performed to assess for persistent CSF leakage before wound closure.

Drains were inserted selectively at the surgeon’s discretion without negative-pressure suction, and wounds were closed in layers.

Postoperative care and follow-up

All patients with incidental dural tears were nursed flat in bed for at least five postoperative days and monitored closely for symptoms suggestive of CSF leakage, including positional headache, nausea, vomiting, diplopia, photophobia, and wound discharge. Follow-up assessments were conducted at six weeks, three months, six months, and one year following surgery. Any postoperative complications, including wound infection, pseudomeningocele formation, or new neurological deficits, were carefully documented.

## Results

A total of 68 patients underwent lumbar spine surgery during the study period and fulfilled the inclusion criteria. Nine patients (13.2%) sustained incidental intraoperative dural tears, all of which were identified and repaired during the index procedure.

Six of the nine patients underwent cyanoacrylate-assisted dural repair, while three were managed using conventional repair techniques without cyanoacrylate. Among the cyanoacrylate-treated patients, four underwent repair using autologous fat grafts and two underwent repair using lumbar fascia grafts.

Intraoperatively, watertight closure was achieved in all six cyanoacrylate-treated cases as assessed by the Valsalva manoeuvre. Postoperatively, one patient developed a superficial wound infection that resolved with antibiotic therapy and local wound care. Another patient, who had undergone revision spine surgery, developed a pseudomeningocele that was successfully managed conservatively without reoperation.

No patient in the cyanoacrylate-treated group developed persistent CSF leakage, meningitis, neurological deterioration, or required re-exploration for dural-related complications. The mean duration of hospital stay among patients with dural tears was 13.7 ± 4.5 days, which was comparable to that of patients without durotomy.

At one-year follow-up, no recurrent CSF leakage or delayed pseudomeningocele formation was observed.

## Discussion

Incidental dural tear remains one of the most frequently encountered complications of lumbar spine surgery, with reported rates between 1% and 17% [1-3]. In our series, the incidence was 13.2%, a figure comparable to reports from other centres in developing countries where degenerative disease often presents late, and tissue planes may be more fibrotic. Early intraoperative recognition and prompt repair remain essential, as missed tears can result in CSF leakage, pseudomeningocele, wound infection, meningitis, and prolonged hospital stay [4,5].

A wide range of repair techniques has been described, ranging from primary suturing with non-absorbable material to the use of autologous grafts such as fat or fascia, and more recently, biologic or synthetic sealants [6-8]. In high-income settings, proprietary products such as fibrin sealants (DuraSeal™ (Integra LifeSciences Corporation, Princeton, New Jersey), Tisseel™ (Baxter Healthcare Corporation, Deerfield, Illinois)) and collagen matrices (DuraGen™ (Integra LifeSciences Corporation), TachoSil™ (Takeda Pharmaceuticals International AG, Zurich, Switzerland)) are routinely used to reinforce closure and minimise CSF leakage [9-11]. In many low- and middle-income environments, however, these materials may be unavailable or prohibitively expensive for routine use. Within this context, lower-cost alternatives may occasionally be explored where clinically appropriate. Cyanoacrylate in our setting costs approximately 0.36 US$ locally compared with commercially available sealants costing between 38 and 120 US$, representing a substantial cost difference.

Cyanoacrylates polymerise rapidly upon contact with tissue moisture to form a seal. Their use in cranial and spinal surgery has been reported with generally satisfactory outcomes, although published experience in dural repair remains relatively limited [12,13]. In our experience, the adhesive was used in six patients who sustained intraoperative dural tears: four repaired with fat grafts and two with fascia grafts. Watertight closure was achieved intraoperatively in all cases, and no patient developed persistent CSF leakage, meningitis, or required re-exploration. One patient developed a mild superficial wound infection that resolved with conservative management, while another developed a pseudomeningocele following revision surgery that was managed without reoperation. These observations are broadly comparable with previous reports describing acceptable short-term outcomes following cyanoacrylate use [14,15].

When compared with fibrin or collagen sealants, cyanoacrylate remains relatively inexpensive and readily available. However, caution should be exercised when interpreting these findings, as the adhesive used in this study was a commercially available ethyl cyanoacrylate product not specifically developed for dural repair. Potential concerns, including inflammatory reactions, tissue toxicity, thermal injury, or brittle sealing, have been described, particularly if applied in excessive quantities or directly over neural tissue [16,17]. In our technique, efforts were made to reduce tissue exposure by applying a limited quantity of adhesive over a biological interface consisting of autologous graft material and Surgicel™ rather than directly onto neural structures.

The findings from this study suggest that cyanoacrylate-assisted repair may represent a feasible low-cost adjunct in carefully selected situations within resource-limited environments. However, the relatively small sample size, retrospective design, absence of comparative analysis, and potential selection bias limit the strength of conclusions that can be drawn. Therefore, definitive conclusions regarding long-term safety, effectiveness, or comparative performance relative to established sealants cannot be made.

This study has some limitations. It was a retrospective review from a single centre with a relatively small number of patients, limiting the strength and generalizability of the findings. There was no direct comparison group using standard commercial sealants, and no formal standardized outcome assessment tool was employed. In addition, follow-up duration may not have been sufficient to detect very late complications.

An additional limitation of this study relates to the routine use of prolonged postoperative flat bed rest for at least five days in patients with incidental dural tears. Although this approach formed part of the standard postoperative management protocol in our setting and was applied irrespective of repair technique, it may itself have contributed to reducing CSF leakage rates. Therefore, it is difficult to isolate the specific contribution of cyanoacrylate reinforcement to the favourable outcomes observed. Furthermore, these findings may not be directly generalisable to centres employing enhanced recovery pathways or early postoperative mobilization protocols.

## Conclusions

Incidental dural tears remain an unavoidable challenge of lumbar spine surgery, even in experienced hands. Early recognition and meticulous intraoperative repair are essential to minimize CSF leakage and its associated complications.

Our experience suggests that cyanoacrylate-assisted repair, when used in combination with autologous fat or fascia grafts and Surgicel™, may represent a feasible and inexpensive adjunct for dural repair in resource-constrained settings. However, these findings should be interpreted cautiously given the small sample size and retrospective design. Larger prospective studies are needed to further evaluate long-term safety, effectiveness, and potential applicability in broader clinical settings.
